# The Canabrava Ring, an open ring-shaped pupil expander for cataract surgery associated with iris colobomata: a case report

**DOI:** 10.5935/0004-2749.20200086

**Published:** 2024-02-11

**Authors:** Sérgio Canabrava, Ana Carolina Canêdo Domingos Lima, Ana Elisa Loyola

**Affiliations:** 1 Departamento de Catarata, Santa Casa de Belo Horizonte, Belo Horizonte, MG, Brazil; 2 Departamento de Catarata, Hospital de Olhos Santa Luzia, Maceió, AL, Brazil

**Keywords:** Phacoemulsification, Equipment design, Pupil, Iris, Coloboma, Facoemulsificação, Desenho de equipamento, Pu pila, Iris, Coloboma

## Abstract

In this report, we describe a new pupil expander device that was used to obtain adequate pupil dilation and centering in a patient with an iris coloboma. Specifically, we describe the case of a patient with an iris coloboma; a Malyugin ring was inserted to facilitate dilation during phacoemulsification surgery. One of the scrolls did not engage which resulted in an uneven distribution of forces and an eccentric pupil. A Canabrava Ring was then implanted that promoted effective pupillary dilation and remained stable and effective throughout the surgical procedure.

## Abstract (pt)

Neste relato, descrevemos um novo dispositivo expansor pupilar que foi usado obter adequada dilatação e centralização da pupila em um paciente com coloboma de íris. Especificamente, descrevemos um caso de cirurgia de facoemulsificação em um paciente com coloboma de íris associado à pupila pequena e que, previamente, tentou-se sem sucesso o uso do expansor Malyugin Ring, que provocou uma dilatação pupilar descentrada. Entretanto, com o uso do expansor de íris Canabrava Ring, a pupila permaneceu dilatada e centrada durante toda a cirurgia, permitindo a realização de um procedimento seguro.

## INTRODUCTION

The term “iris defect” includes any morphological change or lack of structural integrity in this region of the eye. Among these defects are incomplete closures also known as iris colobomata^(1-3)^. Eyes with iris defects are at greater risk for complications during cataract surgery due to poor pupillary dilation combined with an eccentric pupillary aperture^(4)^.

**Figure 1:**
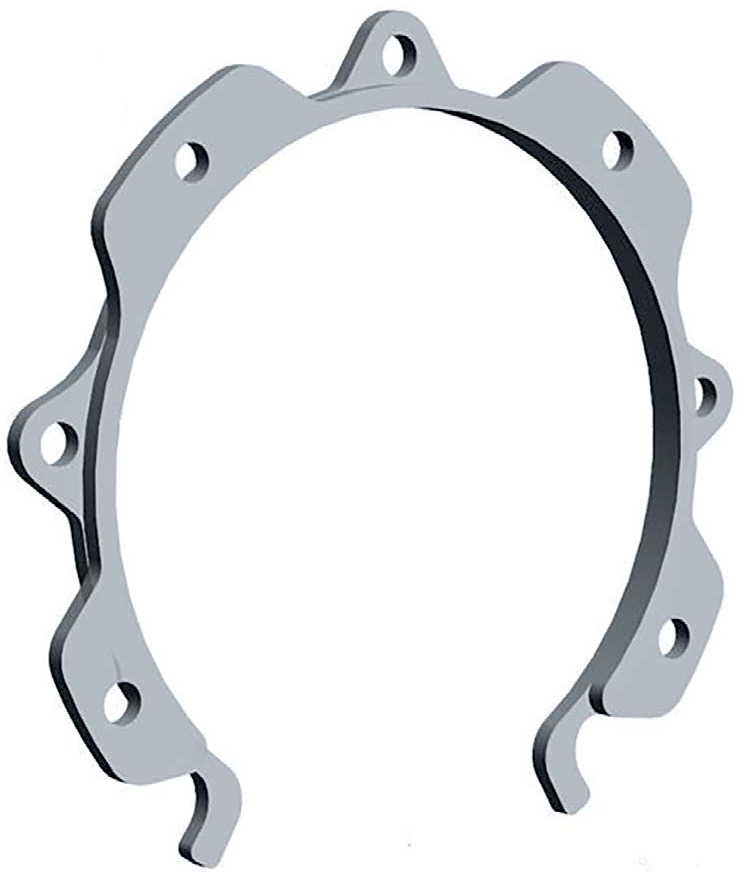
The Canabrava Ring.

Mechanical iris dilators are used during cataract surgery on individuals with small pupils that resist dilation with mydriatics; among these devices are mechanical pupil ring expanders^(5,6)^. However, these mechanical expanders were not formally designed to address the unique surgical challenges associated with iris defects. This paper describes the surgical implantation of a recently developed pupil expander, the Canabrava Ring (AJL Ophthalmic, Miñano, Spain) (Figure 1) in a patient with an iris coloboma and a small pupil.

## CASE REPORT

**Figure 2:**
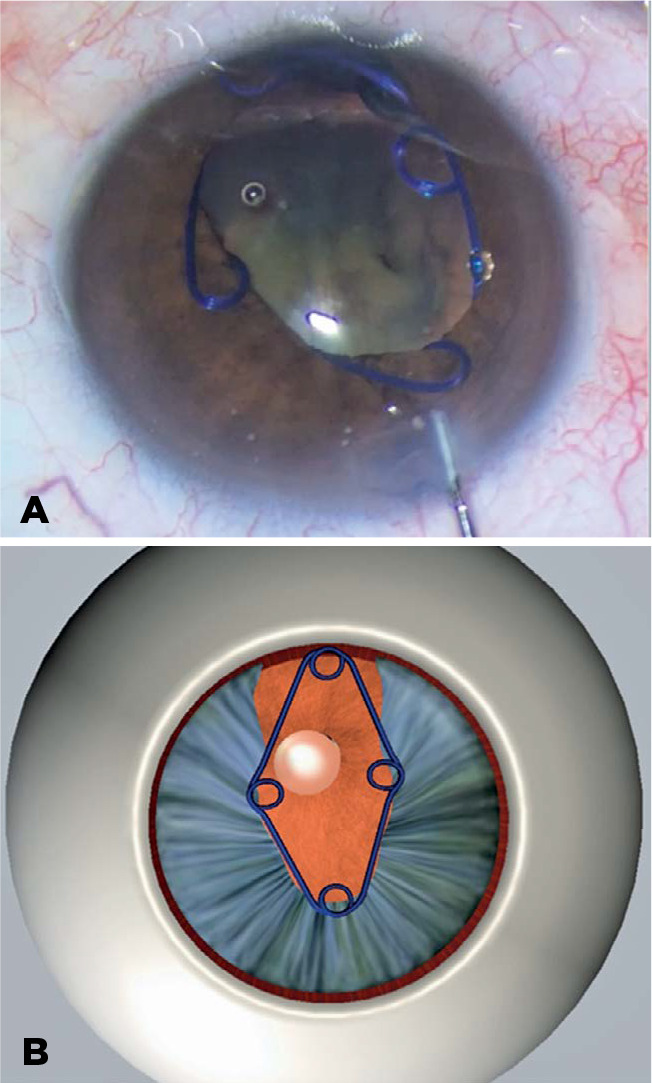
The Malyugin Ring inserted into the congenital iris coloboma.

The patient presented for cataract surgery with an inferior congenital combined iris and lens coloboma with a small pupil. A Malyugin Ring (MTS, Seattle, USA) was inserted to ensure appropriate dilation during the surgical procedure. The device’s scrolls engage the pupil margin and typically result in expansion; however, given the coloboma, one of the scrolls was released, which generated a distribution of forces that moved the ring toward the defect. The pupil was then eccentric and did not undergo proper dilation (Figure 2).

**Figure 3:**
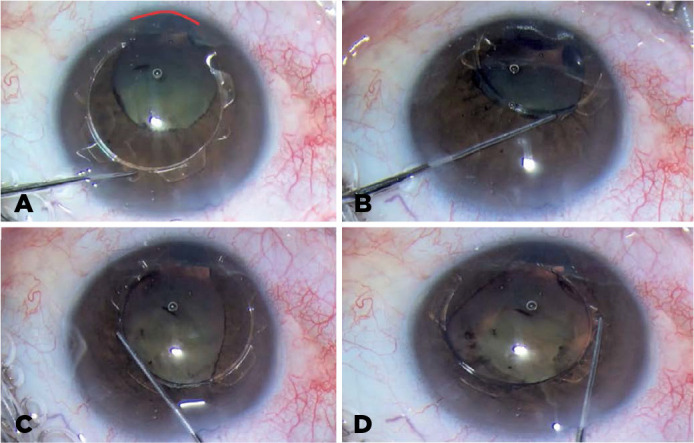
The Canabrava Ring inserted into the congenital iris coloboma.

A 2.2 mm incision was made, and a Canabrava Ring was implanted and docked to the iris. To perform this maneuver, the section to be oriented in the downwards direction was inserted under the edge of the pupil, and then the section to be oriented in the upwards direction was positioned over the pupil edge; the 60° ring opening was aligned with the iris defect, which left the pupil both round and centered (Figure 3). An iris suture was placed at the site of the defect to ensure closure and standard phacoemulsification was performed with the use of an ophthalmic viscosurgical device (OVD); a standard capsular tension ring was inserted to stabilize the capsular bag. The intraocular lens (IOL) was implanted and a second iris suture was made at the coloboma. The Canabrava Ring remained stable during surgery and allowed the procedure to proceed safely and effectively. Phenylephrine was also administered during the procedure. We experienced no post-operative complications.

## DISCUSSION

In cases of iris colobomata, the pupils is oval or slit-shaped and are not capable of effective dilation^(1)^. Several devices have been developed to facilitate pupillary dilation for cataract surgery^(4,7)^. Generally, an iris retractor is a satisfactory option; however, there are several disadvantages associated with this device, including cornea incisions and the instability of the shallow anterior chamber when the iris retractor is fully engaged. Furthermore, most of these retractors are not suitable for use in the case of colobomata, as they require four intact points within the iris to be effective.

As described above, in this case, the Malyugin ring^(8)^ was initially inserted, but one of its scrolls was released which generated forces that left the pupil off-centered (Figure 2). Among several other devices that might be considered, the Visitec I-Ring^(5)^ device latches on to the iris at more points than are used by the Malyugin Ring but, similar to the aforementioned situation, the uneven distribution of forces may end up dilating the pupil into an eccentric position. The B-HEX^®^ Pupil Expander, similar to other expanders that have no aperture, also tends to decentralize the pupil in the presence of an iris coloboma. The Perfect Pupil requires an incision of the cornea of at least 2.8 mm; in the presence of an iris coloboma, this device can only be used in procedures in which the primary incision is coincident with the coloboma site^(9,10)^.

Here, we report successful application of the Canabrava Ring to facilitate pupillary dilation in a patient with an iris coloboma. The Canabrava Ring is a polymethyl methacrylate (PMMA) device with a semi-arch opening of 60°, an internal diameter of 6.3 mm and a vertical length of 0.4 mm. There are seven indentations (0.9 mm horizontal length) which are positioned on the ring in an alternating fashion, including four facing upwards and three facing downwards (Figure 1). These alternating attachments are horizontally aligned and are specifically spaced to facilitate appropriate pupillary dilation when inserted in the iris. Each indentation has a 0.28 mm-wide orifice that facilitates manipulations with a Sinskey Hook. The Canabrava Ring is thin and compact which ensures easy insertion into the ocular globe via an incision as small as 1.4 mm^(10)^.

**Figure 4:**
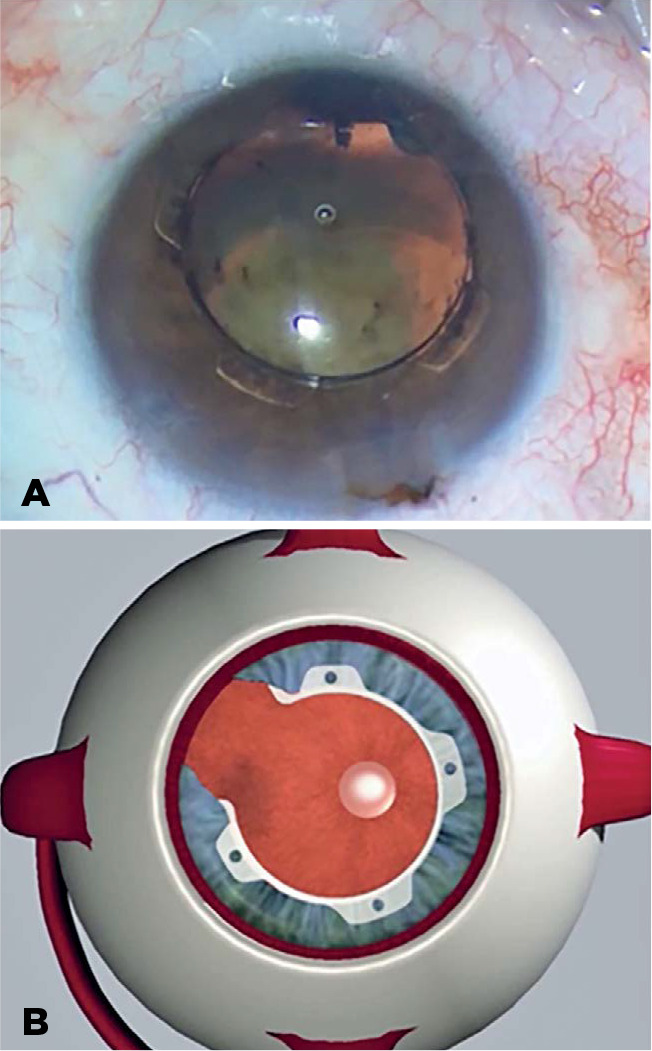
The 60^o^ opening of the Canabrava Ring aligned with the iris coloboma.

To perform the insertion, the opening is first positioned to match the iris defect; this preserves the center of the dilation and provides a uniform distribution of the force vectors regardless of the shape of the coloboma. Second, due to the arrangement of the aforementioned seven alternating indentations, the device can be adjusted and stabilized at the edge of the iris both initially and also during phacoemulsification maneuvers^(10)^. At each end there are two small hooks which attach to the iris; this is particularly useful in cases of iris colobomata, as this permits compensation for the iris defect and the pupil remains centered. The ring attaches to the iris between the superior and inferior indentations, which facilitates stable fixation to the pupil border using the narrowest width possible. Furthermore, as in case of the iris coloboma described here, the 60^o^ opening is particularly helpful as it may be placed in the principal incision axis or in any other direction chosen by the surgeon so that the open edge is aligned with the iris coloboma defect^(10)^ (Figure 4).

In conclusion, the Canabrava Ring is an effective, simple, and non-traumatic solution for managing pupil dilation. Further studies with more patients and longer follow-up periods are needed to determine long-term anatomic outcomes and functional results.
